# Time-Varying Effect of Physical Activity on Mortality Among Myocardial Infarction Survivors: A Nationwide Population-Based Cohort Study

**DOI:** 10.31083/j.rcm2403067

**Published:** 2023-02-22

**Authors:** Teng Li, Xingyi Zhang, Xiuling Wang, Jiali Song, Aoxi Tian, Chaoqun Wu, Xiaoyan Zhang, Yang Yang, Jianlan Cui, Wei Xu, Lijuan Song, Hao Yang, Wenyan He, Yan Zhang, Xi Li, Xin Zheng

**Affiliations:** ^1^National Clinical Research Center for Cardiovascular Diseases, State Key Laboratory of Cardiovascular Disease, Fuwai Hospital, National Center for Cardiovascular Diseases, Chinese Academy of Medical Sciences and Peking Union Medical College, 100037 Beijing, China; ^2^Central China Sub-center of the National Center for Cardiovascular Diseases, 450000 Zhengzhou, Henan, China; ^3^National Clinical Research Center for Cardiovascular Diseases, Shenzhen, Coronary Artery Disease Center, Fuwai Hospital Chinese Academy of Medical Sciences, 518057 Shenzhen, Guangdong, China

**Keywords:** physical activity, myocardial infarction, period, volume, pattern

## Abstract

**Background:**

Physical activity (PA) is an important component of secondary prevention after myocardial infarction (MI). The mortality risk of MI survivors varies at different post-MI periods, yet the time-varying effect of total PA is unclear. We aimed to investigate the association between different volumes and patterns of total PA and mortality at different post-MI periods.

**Methods:**

Using data from the China Patient-centered Evaluative Assessment of Cardiac Events Million Persons Project, we divided the screened MI survivors into within-1-year and beyond-1-year groups based on the duration between their baseline interview and MI onset. Total PA was divided into insufficient (<3000 metabolic equivalent of task [MET] minutes/week) and sufficient PA. Sufficient PA was further categorized as moderate and high (3000–4500 and >4500 MET minutes/week) volumes; leisure (≥50%) and non-leisure (>50%) patterns. Data on mortality were derived from the National Mortality Surveillance System and Vital Registration of the Chinese Center for Disease Control and Prevention. Cox proportional hazard models were fitted to estimate hazard ratios (HRs) and 95% confidence intervals (CIs). Restricted cubic spline regression analyses were performed to examine the dose-response association between PA and mortality.

**Results:**

During the follow-up (median 3.7 years) of the 20,653 post-MI patients, 751 patients died. In the within-1-year group, moderate (HR: 0.59, 95% CI: 0.40 to 0.88) and high (0.63, 0.45 to 0.88) volumes and both patterns (leisure: 0.52, 0.29 to 0.94; non-leisure: 0.64, 0.46 to 0.88) of PA were all associated with significantly lower risk of mortality, compared with insufficient PA. In the beyond-1-year group, the association was observed in high volume (0.69, 0.56 to 0.86) and both patterns (leisure: 0.64, 0.48 to 0.87; non-leisure: 0.79, 0.65 to 0.97). A non-linear relationship between PA and mortality was found in the within-1-year group (*p *for non-linearity <0.001), while a linear relationship was demonstrated in the beyond-1-year group (*p* for non-linearity = 0.107).

**Conclusions:**

Sufficient total PA was associated with mortality risk reduction after MI, either leisure or non-leisure pattern. Different dose-response associations between PA and mortality were found at different post-MI periods. These results could promote individualized and scientifically derived secondary prevention strategies for MI.

## 1. Introduction

In China, the number of patients with myocardial infarction (MI) has been dramatically increasing, and is estimated to reach 23 million by 2030 [1]. MI compromises cardiac function and remodels the cardiac structure, and thus cardiac structure and function may vary in different post-MI periods [2, 3, 4]. The mortality risk of MI survivors is still high [5], and varies at different post-MI periods (within and beyond 1 year) [6]. Physical activity (PA) has been shown to reduce all-cause mortality and cardiovascular mortality of MI patients by 8%–37% and 7%–38%, respectively [7]. Aerobic PA and other lifestyle interventions have been reported to reduce coronary atherosclerosis to different degrees within 1 and 5 years [8]. And PA and exercise-based cardiac rehabilitation have been recommended as important components of secondary prevention of MI and cardiovascular disease (CVD) [9, 10, 11, 12, 13]. The guideline for cardiac rehabilitation in China recommends regular aerobic PA within 1 year after MI and individualized training beyond 1 year after discharge [14]. The possible mechanisms of mortality reduction associated with PA may include improved cardiorespiratory fitness and cardiac remodeling [15]. Thus, we hypothesized that PA may have a different impact on mortality reduction among MI survivors at different post-MI periods.

However, evidence is lacking on the association between different volumes and patterns of total PA and the mortality risks at different post-MI periods. First, there is limited evidence on the association between PA collected at different post-MI periods and mortality among MI survivors. Prior studies primarily used longitudinal cohort follow-up through repeated surveys [16, 17, 18, 19, 20], and focused on PA at 6–10 weeks post-MI [19], or the change of levels of PA after MI [16, 17, 18, 20]. Second, there is a lack of evidence on non-leisure time PA (household, occupational, and transport PA) or total PA (both leisure and non-leisure time PA) among MI patients. Specifically, PA was mainly performed during non-leisure time rather than leisure time in China and other low- and middle-income countries [21]. This differed greatly from those in high-income countries, and the previous evidence mainly focused on the high-income ones [22]. Besides, previous analyses mainly focused on certain populations with small sizes [17, 18, 20]. A comprehensive understanding of the time-varying effect of physical activity on mortality is needed in clinical practice for developing a more tailored strategy for secondary prevention after MI.

We analyzed data from the China Patient-centered Evaluative Assessment of Cardiac Events (PEACE) Million Persons Project (MPP), a population-based cohort study covering 31 provinces in mainland China, to explore the time-varying effect of different volumes and patterns of total PA (including leisure and non-leisure time PA) on mortality risk among MI survivors.

## 2. Materials and Methods

### 2.1 Study Design and Population 

The China PEACE MPP is a government-funded public health project for the screening and management of high CVD risk subjects. The detailed study design has been previously published [23]. From August 2014 to December 2021, permanent residents aged 35–75 years with a history of MI were identified from 349 rural counties or urban districts (210 rural counties, 139 urban districts) in 31 provinces in mainland China. These participants received a detailed questionnaire survey on lifestyle and medical history at baseline, and then were followed up annually (**Supplementary Section 1**). We excluded the participants who reported difficulty in mobility, self-care, or usual activities in the European Quality of Life–5 Dimensions (EQ-5D) questionnaire, as well as those who had incomplete data for PA and covariates (**Supplementary Fig. 1**). Eligible participants were divided into two groups based on the duration between baseline and their MI onset (within and beyond 1 year).

The project protocol was approved by the central ethics committee at Fuwai Hospital, Beijing, China (Approval No. 2014-574). Written informed consent was obtained from all enrolled participants.

### 2.2 Data Collection and Definition 

Data on PA was collected through standardized in-person questionnaire interviews. The questions on PA were adapted from validated questionnaires used in other cohort studies [24, 25, 26]. Participants were asked about detailed information on PA across all domains of life (leisure time, household, occupational, and transport) during the past 12 months. Leisure time PA included swimming, running, or aerobic exercise as vigorous-intensity activities, and ball games, walking, gymnastics, folk dancing, Tai-Chi, and qigong as moderate-intensity activities. Non-leisure time PA included PA in the household, occupational, and transport domains. To quantify the volume of PA, each activity was assigned an intensity level based on the updated 2011 Compendium of Physical Activities [27]. One metabolic equivalent of task (MET) is equivalent to 1 kcal/kg/hour and equal to the energy cost of sitting quietly. The level of each activity was calculated by multiplying its MET value and the minutes spent per week. Domain-specific PA levels were calculated by summing all the MET minutes per week spent in non-sedentary leisure time, household, occupational, and transport domains. Total PA was calculated as a sum of all domain-specific PA (**Supplementary Section 2**).

The theoretical minimum-risk exposure level for total PA was 3000–4500 MET minutes/week recommended by the Global Burden of Disease study [28]. Thus, total PA of less than 3000 MET minutes/week was considered insufficient PA [29]. Sufficient PA was divided into moderate (3000–4500 MET minutes/week) and high (>4500 MET minutes/week) volumes according to the recommendation and categories of the Global Burden of Disease study [28]. In addition, sufficient PA was also further divided into the leisure pattern and non-leisure pattern based on the main domain (leisure time PA ≥50% or non-leisure time PA >50%).

We also collected information on participants’ sociodemographic factors (i.e., age, sex, household income, occupation type, and education level), other lifestyle factors (i.e., diet, smoking, and alcohol consumption), medical history, and medication use during the in-person interviews. Physical measurements (i.e., height, weight, and blood pressure) were collected, and blood lipid and blood glucose levels were determined following standardized protocols by trained medical staff (**Supplementary Section 3**).

### 2.3 Ascertainment of Outcomes

Outcomes of interest in this study were all-cause mortality and cardiovascular mortality, which were identified through the National Mortality Surveillance System and Vital Registration of the Chinese Center for Disease Control and Prevention. All events were coded using the International Classification of Diseases, 10th edition (ICD-10). Cardiovascular mortality was defined as death due to CVD (ICD-10: I01-I99). Mortality data were available up to December 31, 2021. Follow-up was censored at this time or date of death, whichever came first.

### 2.4 Statistical Analysis 

Baseline characteristics were described for the entire cohort, stratified by two post-MI periods and different volumes and patterns of PA. Baseline PA was described for the entire cohort, and the within-1-year and beyond-1-year period. Continuous variables were summarized as means (standard deviations) or medians (interquartile range [IQR]) as appropriate, and categorical variables as frequencies and percentages. Differences in characteristics were examined by one-way analysis of variance for continuous variables and the Chi-square test for categorical variables.

Cox proportional hazard models were fitted to calculate independent hazard ratios (HRs) and 95% confidence intervals (CIs) for the association between different volumes and patterns of PA with all-cause mortality and cardiovascular mortality. We used age instead of time-on-study as the time scale [30, 31, 32]. Multivariable-adjusted models included age, sex, household income, occupation type, education level, high blood pressure, high blood glucose, high total cholesterol, high body mass index, high alcohol consumption, current smoking, unhealthy diet, history of heart failure, history of chronic kidney disease, and medication use including angiotensin-converting enzyme inhibitors or angiotensin receptor blockers, beta-blockers, statins, and aspirin. The interaction between different volumes and patterns of PA and post-MI periods was considered in the statistical models. Using age as the time scale, restricted cubic splines were fitted with 4 knots by treating the volume of total PA as a continuous variable using the “rms” R package. The reference value for PA was set at 3000 MET minutes/week. To reduce potential bias from reverse causation, we also did sensitivity analyses to assess the effect of different volumes and patterns of total PA using inverse probability weighting. Specifically, we included the participants who reported difficulty in mobility, self-care, or usual activities in the EQ-5D questionnaire, and calculated the propensity scores based on the EQ-5D score and covariates of the multivariable-adjusted models. Then the Cox proportional hazards models were weighted to inverse probability weighting.

All analyses were performed in SAS 9.4 (SAS Institute Inc., Cary, NC, USA) and R software, version 4.1.2 (The R Foundation for Statistical Computing, Vienna, Austria).

## 3. Results

### 3.1 Baseline Characteristics

We enrolled 29,913 participants with prior MI in the China PEACE MPP. After excluding participants with difficulty in mobility, self-care, and usual activities (1293, 4.3%), and those with incomplete information on PA (5463, 19.1%) and covariates (2504, 10.8%), 20,653 participants were finally included (**Supplementary Fig. 1**). Among the included patients, the median age was 62 years and 47.7% were female (Table 1). Patient characteristics differed in most features across the two post-MI periods, except for sex (*p = *0.620), history of chronic kidney disease (*p = *0.928), statins use (*p = *0.499), and aspirin use (*p = *0.109). Baseline characteristics were also stratified by different volumes and patterns of PA (**Supplementary Table 1**). Baseline PA at each post-MI period is presented in Table 2. MI patients in the within-1-year group had a higher volume of total PA (median 4200 versus 3780 MET minutes/week, *p <* 0.001) and non-leisure time PA (median 3360 versus 2384 MET minutes/week, *p <* 0.001) compared to those in the beyond-1-year group. Total PA was also categorized into insufficient and sufficient PA; sufficient PA was further categorized as moderate and high by volumes, and leisure and non-leisure by domains. There were 1802 (34.5%) patients who reported insufficient total PA in the within-1-year group, and 6003 (38.9%) in the beyond-1-year group. Non-leisure time PA had a larger proportion in both time periods (56.9% within 1 year, 50.0% beyond 1 year); while the numbers of patients having leisure pattern were small (8.6% within 1 year, 11.1% beyond 1 year).

**Table 1.** **Baseline characteristics of the participants stratified by post-MI periods**.

Characteristics			Total period	Within 1 year of MI onset	Beyond 1 year of MI onset	*p-*value
Number of participants			20,653	5222 (25.3%)	15,431 (74.7%)	
Socioeconomic factors						
	Age (years)		62 [55, 67]	61 [53, 66]	63 [56, 68]	<0.001
	Sex					
		Female	9854 (47.7%)	2507 (48.0%)	7347 (47.6%)	0.620
		Male	10,799 (52.3%)	2715 (52.0%)	8084 (52.4%)	
	Household income					
		<10,000 (yuan/year)	3880 (18.8%)	1073 (20.5%)	2807 (18.2%)	<0.001
		≥10,000 (yuan/year)	15,340 (74.3%)	3764 (72.1%)	11,576 (75.0%)	
		Unknown	1433 (6.9%)	385 (7.4%)	1048 (6.8%)	
	Occupation type					
		Light intensity	2734 (13.2%)	745 (14.3%)	1989 (12.9%)	<0.001
		Medium or heavy intensity	2973 (14.4%)	912 (17.5%)	2061 (13.4%)	
		Unemployed or retired	11,690 (56.6%)	2676 (51.2%)	9014 (58.4%)	
		Unknown	3256 (15.8%)	889 (17.0%)	2367 (15.3%)	
	Education level					
		Primary school or below	8557 (41.4%)	2256 (43.2%)	6301 (40.8%)	0.011
		Middle school or above	11,918 (57.7%)	2921 (55.9%)	8997 (58.3%)	
		Unknown	178 (0.9%)	45 (0.9%)	133 (0.9%)	
Metabolic factors						
	High blood pressure		10,419 (50.4%)	2436 (46.6%)	7983 (51.7%)	<0.001
	High blood glucose		5108 (24.7%)	1181 (22.6%)	3927 (25.4%)	<0.001
	High total cholesterol		5565 (26.9%)	1247 (23.9%)	4318 (28.0%)	<0.001
	High body mass index		11,400 (55.2%)	2801 (53.6%)	8599 (55.7%)	0.009
Lifestyle factors						
	Current smoking		4789 (23.2%)	1150 (22.0%)	3639 (23.6%)	0.021
	High alcohol consumption		2127 (10.3%)	467 (8.9%)	1660 (10.8%)	<0.001
	Unhealthy diet		17,991 (87.1%)	4596 (88.0%)	13,395 (86.8%)	0.025
Medical History						
	Heart failure		948 (4.6%)	209 (4.0%)	739 (4.8%)	0.019
	Chronic kidney disease		188 (0.9%)	47 (0.9%)	141 (0.9%)	0.928
Medication use						
	ACEIs or ARBs		2441 (11.8%)	540 (10.3%)	1901 (12.3%)	<0.001
	Beta-blockers		2073 (10.0%)	428 (8.2%)	1645 (10.7%)	<0.001
	Statins		3105 (15.0%)	770 (14.8%)	2335 (15.1%)	0.499
	Aspirin		3442 (16.7%)	833 (16.0%)	2609 (16.9%)	0.109

Data are presented as mean (SD), median [Q1, Q3 quartiles], or n (%) as appropriate. ACEIs, angiotensin-converting enzyme inhibitors; ARBs, angiotensin receptor blockers; MET, metabolic equivalent of task; MI, myocardial infarction; PA, physical activity; SD, standard deviation.

**Table 2.** **Baseline PA of the participants stratified by post-MI periods**.

PA (MET minutes/week)		Total period	Within 1 year of MI onset	Beyond 1 year of MI onset	*p*-value
Domains of PA					
	Total PA	3948 [2352, 6930]	4200 [2352, 7336]	3780 [2352, 6741]	<0.001
	Leisure time PA	0 [0, 1386]	0 [0, 1386]	0 [0, 1386]	<0.001
	Non-Leisure time PA	2520 [1176, 5880]	3360 [1440, 6544]	2384 [1176, 5760]	<0.001
Volumes of total PA					
	Insufficient (<3000)	7805 (37.8%)	1802 (34.5%)	6003 (38.9%)	<0.001
	Moderate (3000–4500)	3772 (18.3%)	952 (18.2%)	2820 (18.3%)	
	High (>4500)	9076 (43.9%)	2468 (47.3%)	6608 (42.8%)	
Patterns of total PA					
	Insufficient (<3000)	7805 (37.8%)	1802 (34.5%)	6003 (38.9%)	<0.001
	Non-leisure (main domain)	10,686 (51.7%)	2971 (56.9%)	7715 (50.0%)	
	Leisure (main domain)	2162 (10.5%)	449 (8.6%)	1713 (11.1%)	

Data are presented as mean (SD), median [Q1, Q3 quartiles], or n (%) as appropriate. Total PA (MET minutes/week) was categorized as: insufficient (<3000); moderate (3000–4500) and high (>4500) volumes; non-leisure (sufficient PA with more input from non-leisure time PA) and leisure patterns (sufficient PA with more input from leisure time PA). MET, metabolic equivalent of task; MI, myocardial infarction; PA, physical activity; SD, standard deviation.

### 3.2 Health Benefits of Different Volumes of PA

**Fig. 1.**
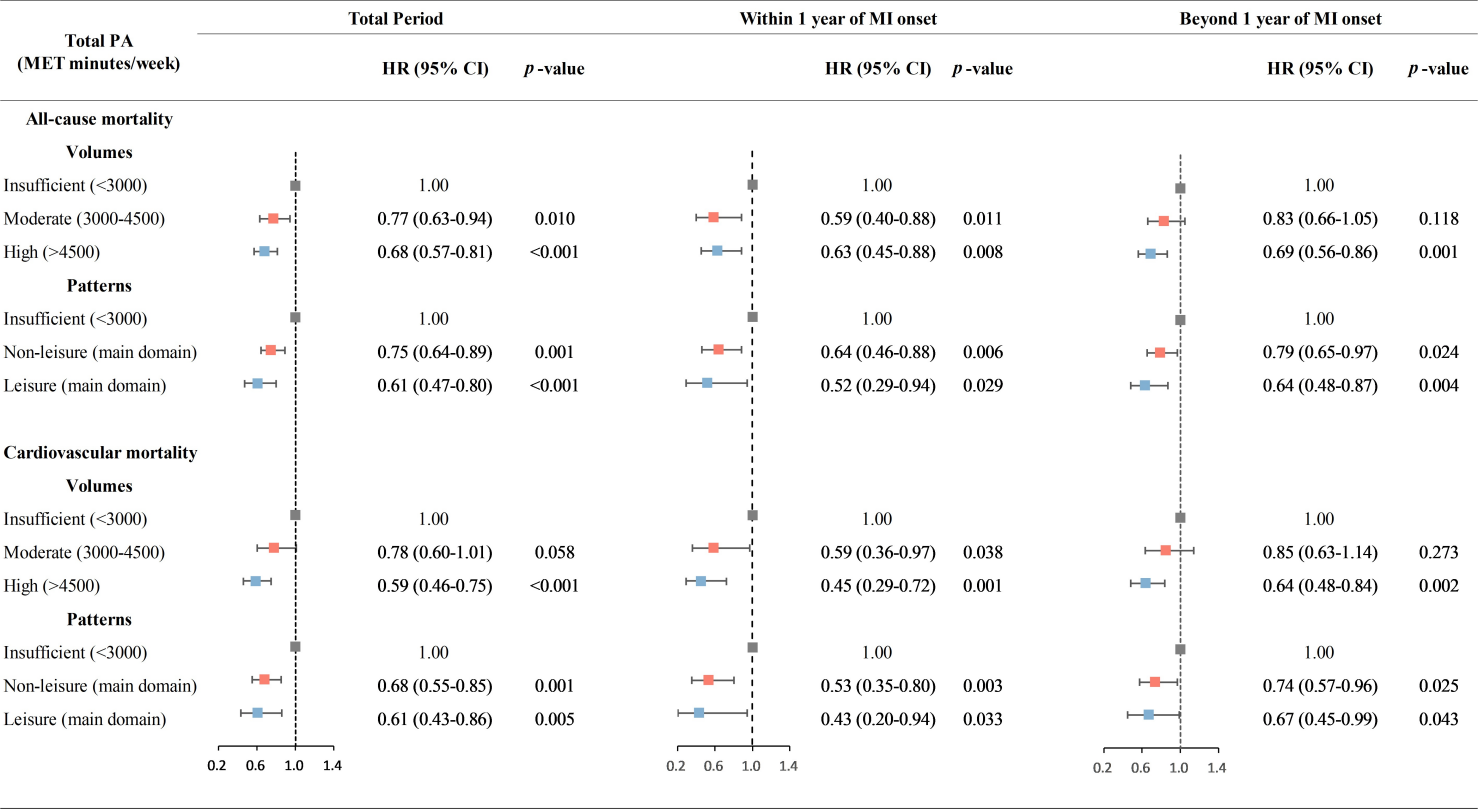
**HRs (95% CI) for all-cause mortality and cardiovascular mortality by PA volumes at different post-MI periods**. Total PA (MET minutes/week) was categorized as: insufficient (<3000); moderate (3000–4500) and high (>4500) volumes; non-leisure (sufficient PA with more input from non-leisure time PA) and leisure (sufficient PA with more input from leisure time PA) patterns. HRs were adjusted for age, sex, household income, occupation type, education level, high blood pressure, high blood glucose, high total cholesterol, high body mass index, high alcohol consumption, current smoking, unhealthy diet, history of heart failure, history of chronic kidney disease, and medication use including angiotensin-converting enzyme inhibitors or angiotensin receptor blockers, beta-blockers, statins, and aspirin. CI, confidence interval; HR, hazard ratio; MET, metabolic equivalent of task; MI, myocardial infarction; PA, physical activity.

During the median follow-up of 3.7 years (IQR 2.6–4.8 years), there were 751 all-cause deaths, including 446 cardiovascular deaths (**Supplementary Table 2**). The associations between different volumes of PA and mortality before and after adjusting for covariates are displayed in **Supplementary Table 3** and Fig. 1, respectively.

**Fig. 2.**
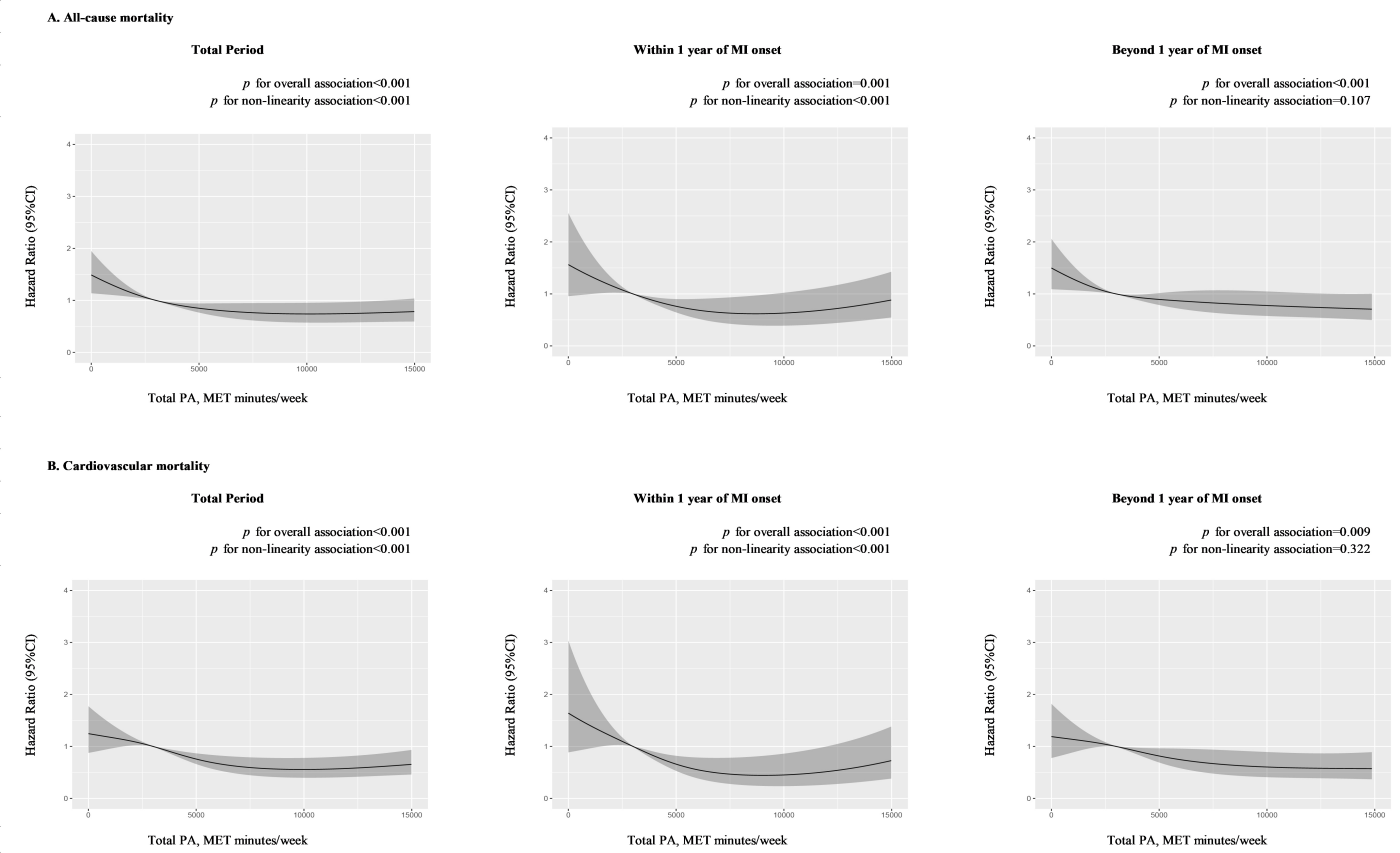
**Dose-response association between PA and mortality at different post-MI periods**. (A) Dose-response association between total PA and all-cause mortality at total, within-1-year, and beyond-1-year period. (B) Dose-response association between total PA and cardiovascular mortality at total, within-1-year, and beyond-1-year period. Hazard ratios were adjusted for age, sex, household income, occupation type, education level, high blood pressure, high blood glucose, high total cholesterol, high body mass index, high alcohol consumption, current smoking, unhealthy diet, history of heart failure, history of chronic kidney disease, and medication use including angiotensin-converting enzyme inhibitors or angiotensin receptor blockers, beta-blockers, statins, and aspirin. MET, metabolic equivalent of task; MI, myocardial infarction; PA, physical activity.

Among the survivors within 1 year of MI onset, moderate PA was associated with significantly lower risk of mortality both in unadjusted analyses (HR 0.59, 95% CI 0.40 to 0.88, *p* = 0.009) and fully adjusted models (0.59, 0.40 to 0.88, *p* = 0.011), while a significant association between high PA and mortality was only observed in fully adjusted models (0.63, 0.45 to 0.88, *p* = 0.008), compared to those with insufficient PA. As shown in Fig. 2, the risk of mortality showed an inverse non-linear relationship with the volume of total PA (*p* for non-linearity < 0.001).

Among the survivors beyond 1 year of MI onset, moderate PA was not significantly associated with lower mortality risk after adjusting for covariates (unadjusted: 0.79, 0.62 to 0.99, *p = *0.042; adjusted: 0.83, 0.66 to 1.05, *p = *0.118), while HRs of high PA were significant both before (0.70, 0.58 to 0.85) and after (0.69, 0.56 to 0.86) adjusting for covariates. The dose-response association (Fig. 2) between total PA and mortality risk was linear in the beyond-1-year group (*p* for non-linearity = 0.107).

No significant interaction was found between different volumes and post-MI periods (*p = *0.515 for interaction). Similar findings were also observed between different volumes of PA and cardiovascular mortality. No substantial changes in the results were observed in the sensitivity analyses using the inverse probability weighting method (**Supplementary Table 4**).

### 3.3 Health Benefits of Different Patterns of PA

Overall, sufficient PA, i.e., leisure and non-leisure patterns, was associated with significantly lower risk of mortality compared with those with insufficient PA at each post-MI period.

In the unadjusted models (**Supplementary Table 5**), both leisure and non-leisure patterns were associated with significantly lower HRs for all-cause mortality in within-1-year (leisure: 0.46, 0.26 to 0.82; non-leisure: 0.74, 0.56 to 0.98) and beyond-1-year (leisure: 0.61, 0.46 to 0.83; non-leisure: 0.77, 0.64 to 0.92) groups. In the fully adjusted models (Fig. 1, **Supplementary Table 5**), among survivors within 1 year of MI onset, leisure (0.52, 0.29 to 0.94) and non-leisure (0.64, 0.46 to 0.88) patterns were both associated with significantly lower risk of mortality. A similar association was observed among survivors beyond 1 year of MI onset (leisure: 0.64, 0.48 to 0.87; nonleisure: 0.79, 0.65 to 0.97). However, no significant interaction was found between different patterns of PA and post-MI periods (*p = *0.775 for interaction). The results were consistent for cardiovascular mortality, and did not change substantially in the sensitivity analyses using the inverse probability weighting method (**Supplementary Table 6**).

## 4. Discussion

Our study found that sufficient total PA was associated with lower risk of mortality among MI survivors. The dose-response association of total PA and mortality was curvilinear within 1 year and linear after 1 year of MI onset. The reduction in mortality risk among MI survivors appeared to be apparent with moderate PA within 1 year after MI, and those with high PA beyond 1 year after MI. Both leisure and non-leisure patterns were associated with lower mortality risk, independent of the post-MI periods.

This study adds to the prior literature in several ways. First, we provided information regarding the association between total PA and mortality risk among MI survivors and found a difference between the within-1-year and beyond-1-year groups. Guidelines for cardiac rehabilitation recommend regular moderate-intensity aerobic PA lasting 30–60 minutes on each day and 3–5 days/week for stage 2 rehabilitation (within 1 year after discharge), and an individual home-based training program for stage 3 rehabilitation (beyond 1 year after discharge) [14]. One possible reason for the different recommendations is the potential heterogeneity in cardiac structure and function [3, 4], and mortality risk [5] between two post-MI periods. In this study, a significant difference in the distribution of PA was observed among MI survivors within and beyond 1 year of MI onset. Within 1 year, MI survivors tended to perform less leisure time PA, compared with those beyond 1 year. It is a reasonable assumption that MI survivors within 1 year tended to be reluctant to perform leisure time PA for fear of adverse events following their acute MI. However, although they did not actively select leisure time PA, they performed more non-leisure time PA and total PA, and had a lower proportion of insufficient PA. Apart from the possible different cardiac structures and functions, we speculate that the difference in the association between PA and mortality risk at two post-MI periods might be partly due to the distribution of PA habits.

Second, we report the association between different volumes of PA and the risk of mortality at different post-MI periods. Previous studies of graded effects of total PA were limited to the general population and patients with CVD [22, 33]. Although the Global Burden of Disease study recommends 3000–4500 MET minutes/week of total PA [28] based on a meta-analysis quantifying the dose-response associations in five chronic diseases (including ischemic heart disease events) [34], it is still unclear whether a similar volume could also work in MI survivors at different post-MI periods. In our study, >3000 MET minutes/week of total PA was associated with lower mortality risk in the within-1-year group, while in the beyond-1-year period, a higher volume (>4500 MET minutes/week) of total PA was associated with lower risk of mortality. This curvilinear association was found in patients within 1 year of MI onset, with a steep risk reduction at a moderate volume of total PA and risk reduction plateauing at higher volumes, which suggested no additional health benefits above a certain volume of total PA. The linear relationship in the beyond-1-year group indicates that performing PA at a higher volume was consistently associated with lower risk of mortality. The potential explanation for the different shapes of dose-response association in different post-MI periods is not clear. Previous studies reported a similar non-linear shape of the dose-response association between PA and mortality in individuals without CVD [33] and in those with stable coronary heart disease [35], while a the linear relationship was reported in CVD patients [33]. Previous evidence showed that vigorous PA transiently increased the risk for sudden cardiac death [36], and vigorous intensities and large PA volumes were both associated with potential accelerated coronary artery calcification, exercise-induced cardiac biomarker release, myocardial fibrosis, and atrial fibrillation [37]. We speculate that cardiac structure and function are not sufficiently recovered within 1 year of MI onset, and that the difference in cardiac structure and function may relate to the different dose-response associations.

Third, our study filled the gap in the association between different patterns of sufficient PA and mortality risk. We found that sufficient total PA, either leisure (higher proportion of leisure time PA) or non-leisure (higher proportion of non-leisure time PA) pattern, was associated with lower risk of mortality, compared with insufficient total PA. In our study, only a few MI patients had leisure pattern. This is consistent with previous studies which observed that PA in China mainly involved occupation and housework, with less contribution from leisure time PA [21]. Prior evidence on leisure time PA [33, 38] and non-leisure time PA [22, 33] was reported in the general population and patients with CVD, among which the evidence was inconsistent on non-leisure time PA. And in the research where non-leisure time PA was categorized into the domains of household, occupational, and transport, the findings were still inconsistent [39, 40, 41, 42]. Leisure time PA is usually a short period PA with a long recovery time, which may improve cardiovascular risk factors (such as insulin resistance, hypertension, dyslipidemia, and obesity) [40, 43]. However, non-leisure time PA, such as occupational PA, is of low intensity or long duration and may not improve cardiovascular health. Occupational PA could raise 24-hour heart rate and blood pressure, and increase the levels of inflammation [41]. While the potential mechanisms of household and transport PA still need further study. The possible influential factors are that some housework is also an aerobic PA with sufficient recovery time, while transport PA may be affected by traffic and harmful exposure (e.g., gases on roads) as well as active or passive commuting types [42].

Our findings have several important implications. First, appropriately high PA after MI indicates lower risk of mortality. This finding serves as empirical support for making scientifically warranted secondary prevention recommendations for the post-MI population to promote sufficient PA. Second, the evidence on proper volumes and patterns of PA for patients at different post-MI periods could facilitate more tailored health advice for PA after MI. Third, for MI survivors within 1 year of their MI onset, as non-leisure time PA is usually performed on a daily basis, we suggest selecting an adequate volume of leisure time PA after fully considering their non-leisure time PA to reach a total level of approximately at least 3000 MET minutes/week within 1 year of MI onset, and no less than 4500 MET minutes/week beyond 1 year of MI onset. Nevertheless, patients should be cautious about their choice in the proper volume of PA. The volumes well above the PA recommendations were reported to be associated with increased mortality risk in CVD patients [44, 45]. More importantly, MI patients should consult with their physicians before adjusting for their PA volume.

Our study has several limitations. First, MI history and PA information were self-reported, which may bring recall bias and may overestimate PA volume [46]. However, questionnaires were collected by trained professionals following a standardized process, the self-reported medical information was based on physician diagnosis, and the questions of PA information had been used and validated in other cohort studies [24, 25, 26]. We assumed that the recall bias is relatively small in a large-scale population. Second, we used the recommended total PA level of the Global Burden of Disease study rather than the widely used World Health Organization guideline [47]. In Chinese adults, PA mainly involves occupation and housework, without much input from leisure time PA [21], so the studies in China usually referred to the World Health Organization standard for leisure time PA rather than total PA [48]. Third, the study lacks data on important clinical risk factors, such as exercise capacity, cardiac function, and disease severity, which are also potential confounders. Nevertheless, we adjusted for history of heart failure, history of chronic kidney disease, metabolic factors, lifestyle factors, and medication use to reduce the confounding effect when analyzing the association between PA and mortality. Fourth, a risk of reverse causality is present, so participants with less severity of MI may have a higher PA and a better prognosis. To address the concerns related to reverse causality, we excluded the participants who reported problems in walking, self-care, and daily activities in the EQ-5D questionnaire. In addition, we did sensitivity analyses using the inverse probability weighting method, and the results did not substantially change our main analyses. However, reverse causality may be unavoidable in observational studies, establishing the optimal volume and pattern of PA in each clinical setting would require randomized head-to-head comparisons.

## 5. Conclusions

Our findings demonstrated that sufficient total PA was associated with lower risk of mortality compared with insufficient PA among MI survivors. The dose-response association varied by the different post-MI periods. Both leisure and non-leisure patterns were associated with lower mortality risk, independent of the post-MI periods. These findings could facilitate tailored recommendations on PA in secondary prevention after MI. Further studies are needed to confirm the causal effect of different volumes and patterns of PA on mortality risk at different periods after MI.

## Data Availability

The data that support the findings of this study are available from China Patient-centered Evaluative Assessment of Cardiac Events Million Persons Project (China PEACE MPP) but restrictions apply to the availability of these data, which were used under license for the current study, and so are not publicly available. Data are however available from the authors upon reasonable request and with permission of China PEACE MPP. China PEACE MPP only provides conditional data access for qualified researchers with legitimate requests; a formal application and research proposal is required. Please contact cvd-project@nccd.org.cn to seek approval for data access.

## Abbreviations

CVD, cardiovascular disease; China PEACE MPP, China Patient-centered Evaluative Assessment of Cardiac Events Million Persons Project; CI, confidence interval; HR, hazard ratio; ICD-10, International Classification of Diseases, 10th edition; IQR, interquartile range; MI, myocardial infarction; MET, metabolic equivalent of task; PA, physical activity; SD, standard deviation.

## Supplementary Material

Supplementary material associated with this article can be found, in the online version, at https://doi.org/10.31083/j.rcm2403067.

## References

[1] The World Bank (2011). Toward a healthy and harmonious life in China: stemming the rising tide of non-communicable diseases. http://www.worldbank.org/content/dam/Worldbank/document/NCD_report_en.pdf.

[2] Zhang F, Hu G, Chen X, Zhang L, Guo L, Li C (2022). Excessive branched-chain amino acid accumulation restricts mesenchymal stem cell-based therapy efficacy in myocardial infarction. *Signal Transduction and Targeted Therapy*.

[3] Cohn JN, Ferrari R, Sharpe N (2000). Cardiac remodeling–concepts and clinical implications: a consensus paper from an international forum on cardiac remodeling. Behalf of an International Forum on Cardiac Remodeling. *Journal of the American College of Cardiology*.

[4] Yalta K, Yilmaz MB, Yalta T, Palabiyik O, Taylan G, Zorkun C (2020). Late Versus Early Myocardial Remodeling After Acute Myocardial Infarction: A Comparative Review on Mechanistic Insights and Clinical Implications. *Journal of Cardiovascular Pharmacology and Therapeutics*.

[5] Jernberg T, Hasvold P, Henriksson M, Hjelm H, Thuresson M, Janzon M (2015). Cardiovascular risk in post-myocardial infarction patients: nationwide real world data demonstrate the importance of a long-term perspective. *European Heart Journal*.

[6] Norgaard ML, Andersen SS, Schramm TK, Folke F, Jørgensen CH, Hansen ML (2010). Changes in short- and long-term cardiovascular risk of incident diabetes and incident myocardial infarction–a nationwide study. *Diabetologia*.

[7] Oldridge NB, Guyatt GH, Fischer ME, Rimm AA (1988). Cardiac rehabilitation after myocardial infarction. Combined experience of randomized clinical trials. *Journal of the American Medical Association*.

[8] Ornish D, Scherwitz LW, Billings JH, Brown SE, Gould KL, Merritt TA (1998). Intensive lifestyle changes for reversal of coronary heart disease. *Journal of the American Medical Association*.

[9] Ibanez B, James S, Agewall S, Antunes MJ, Bucciarelli-Ducci C, Bueno H (2018). 2017 ESC Guidelines for the management of acute myocardial infarction in patients presenting with ST-segment elevation: The Task Force for the management of acute myocardial infarction in patients presenting with ST-segment elevation of the European Society of Cardiology (ESC). *European Heart Journal*.

[10] O’Gara PT, Kushner FG, Ascheim DD, Casey DE, Chung MK, de Lemos JA (2013). 2013 ACCF/AHA guideline for the management of ST-elevation myocardial infarction: a report of the American College of Cardiology Foundation/American Heart Association Task Force on Practice Guidelines. *Circulation*.

[11] Collet J, Thiele H, Barbato E, Barthélémy O, Bauersachs J, Bhatt DL (2021). 2020 ESC Guidelines for the management of acute coronary syndromes in patients presenting without persistent ST-segment elevation. *European Heart Journal*.

[12] Knuuti J, Wijns W, Saraste A, Capodanno D, Barbato E, Funck-Brentano C (2020). 2019 ESC Guidelines for the diagnosis and management of chronic coronary syndromes. *European Heart Journal*.

[13] Pelliccia A, Sharma S, Gati S, Bäck M, Börjesson M, Caselli S (2021). 2020 ESC Guidelines on sports cardiology and exercise in patients with cardiovascular disease. *European Heart Journal*.

[14] (2021). Guideline for primary care of cardiac rehabilitation of coronary artery disease (2020). *Chinese Journal of General Practitioners of Chinese Medical Association*.

[15] Tucker WJ, Fegers-Wustrow I, Halle M, Haykowsky MJ, Chung EH, Kovacic JC (2022). Exercise for Primary and Secondary Prevention of Cardiovascular Disease: JACC Focus Seminar 1/4. *JACC: Journal of the American College of Cardiology*.

[16] Ekblom O, Ek A, Cider Å, Hambraeus K, Börjesson M (2018). Increased Physical Activity Post-Myocardial Infarction Is Related to Reduced Mortality: Results From the SWEDEHEART Registry. *Journal of the American Heart Association*.

[17] Steffen-Batey L, Nichaman MZ, Goff DC, Frankowski RF, Hanis CL, Ramsey DJ (2000). Change in level of physical activity and risk of all-cause mortality or reinfarction: The Corpus Christi Heart Project. *Circulation*.

[18] Gorczyca AM, Eaton CB, LaMonte MJ, Manson JE, Johnston JD, Bidulescu A (2017). Change in Physical Activity and Sitting Time After Myocardial Infarction and Mortality Among Postmenopausal Women in the Women’s Health Initiative-Observational Study. *Journal of the American Heart Association*.

[19] Ek A, Ekblom Ö, Hambraeus K, Cider Å, Kallings LV, Börjesson M (2019). Physical inactivity and smoking after myocardial infarction as predictors for readmission and survival: results from the SWEDEHEART-registry. *Clinical Research in Cardiology: Official Journal of the German Cardiac Society*.

[20] Al-Shaar L, Li Y, Rimm EB, Manson JE, Rosner B, Hu FB (2020). Physical Activity and Mortality among Male Survivors of Myocardial Infarction. *Medicine and Science in Sports and Exercise*.

[21] Du H, Li L, Whitlock G, Bennett D, Guo Y, Bian Z (2014). Patterns and socio-demographic correlates of domain-specific physical activities and their associations with adiposity in the China Kadoorie Biobank study. *BMC Public Health*.

[22] Lear SA, Hu W, Rangarajan S, Gasevic D, Leong D, Iqbal R (2017). The effect of physical activity on mortality and cardiovascular disease in 130 000 people from 17 high-income, middle-income, and low-income countries: the PURE study. *Lancet*.

[23] Lu J, Xuan S, Downing NS, Wu C, Li L, Krumholz HM (2016). Protocol for the China PEACE (Patient-centered Evaluative Assessment of Cardiac Events) Million Persons Project pilot. *BMJ Open*.

[24] Chen Z, Chen J, Collins R, Guo Y, Peto R, Wu F (2011). China Kadoorie Biobank of 0.5 million people: survey methods, baseline characteristics and long-term follow-up. *International Journal of Epidemiology*.

[25] Matthews CE, Shu X, Yang G, Jin F, Ainsworth BE, Liu D (2003). Reproducibility and validity of the Shanghai Women’s Health Study physical activity questionnaire. *American Journal of Epidemiology*.

[26] Wareham NJ, Jakes RW, Rennie KL, Mitchell J, Hennings S, Day NE (2002). Validity and repeatability of the EPIC-Norfolk Physical Activity Questionnaire. *International Journal of Epidemiology*.

[27] Ainsworth BE, Haskell WL, Herrmann SD, Meckes N, Bassett DR, Tudor-Locke C (2011). 2011 Compendium of Physical Activities: a second update of codes and MET values. *Medicine and Science in Sports and Exercise*.

[28] GBD 2017 Risk Factor Collaborators (2018). Global, regional, and national comparative risk assessment of 84 behavioural, environmental and occupational, and metabolic risks or clusters of risks for 195 countries and territories, 1990-2017: a systematic analysis for the Global Burden of Disease Study 2017. *Lancet*.

[29] Li X, Wu C, Lu J, Chen B, Li Y, Yang Y (2020). Cardiovascular risk factors in China: a nationwide population-based cohort study. *The Lancet Public Health*.

[30] Thiébaut ACM, Bénichou J (2004). Choice of time-scale in Cox’s model analysis of epidemiologic cohort data: a simulation study. *Statistics in Medicine*.

[31] Canchola AJ, Stewart SL, Bernstein L, West DW, Ross RK, Deapen D (2003). Cox regression using different time-scales. https://core.ac.uk/display/101895398.

[32] Korn EL, Graubard BI, Midthune D (1997). Time-to-event analysis of longitudinal follow-up of a survey: choice of the time-scale. *American Journal of Epidemiology*.

[33] Bakker EA, Lee D, Hopman MTE, Oymans EJ, Watson PM, Thompson PD (2021). Dose-response association between moderate to vigorous physical activity and incident morbidity and mortality for individuals with a different cardiovascular health status: A cohort study among 142,493 adults from the Netherlands. *PLoS Medicine*.

[34] Kyu HH, Bachman VF, Alexander LT, Mumford JE, Afshin A, Estep K (2016). Physical activity and risk of breast cancer, colon cancer, diabetes, ischemic heart disease, and ischemic stroke events: systematic review and dose-response meta-analysis for the Global Burden of Disease Study 2013. *BMJ*.

[35] Stewart RAH, Held C, Hadziosmanovic N, Armstrong PW, Cannon CP, Granger CB (2017). Physical Activity and Mortality in Patients With Stable Coronary Heart Disease. *Journal of the American College of Cardiology*.

[36] Albert CM, Mittleman MA, Chae CU, Lee IM, Hennekens CH, Manson JE (2000). Triggering of sudden death from cardiac causes by vigorous exertion. *The New England Journal of Medicine*.

[37] Franklin BA, Thompson PD, Al-Zaiti SS, Albert CM, Hivert M, Levine BD (2020). Exercise-Related Acute Cardiovascular Events and Potential Deleterious Adaptations Following Long-Term Exercise Training: Placing the Risks Into Perspective-An Update: A Scientific Statement From the American Heart Association. *Circulation*.

[38] Jeong S, Kim S, Kang S, Kim H, Yoon C, Youn T (2019). Mortality reduction with physical activity in patients with and without cardiovascular disease. *European Heart Journal*.

[39] Autenrieth CS, Baumert J, Baumeister SE, Fischer B, Peters A, Döring A (2011). Association between domains of physical activity and all-cause, cardiovascular and cancer mortality. *European Journal of Epidemiology*.

[40] Nystoriak MA, Bhatnagar A (2018). Cardiovascular Effects and Benefits of Exercise. *Frontiers in Cardiovascular Medicine*.

[41] Holtermann A, Krause N, van der Beek AJ, Straker L (2018). The physical activity paradox: six reasons why occupational physical activity (OPA) does not confer the cardiovascular health benefits that leisure time physical activity does. *British Journal of Sports Medicine*.

[42] Patterson R, Panter J, Vamos EP, Cummins S, Millett C, Laverty AA (2020). Associations between commute mode and cardiovascular disease, cancer, and all-cause mortality, and cancer incidence, using linked Census data over 25 years in England and Wales: a cohort study. *The Lancet Planetary Health*.

[43] Pedersen BK, Saltin B (2015). Exercise as medicine - evidence for prescribing exercise as therapy in 26 different chronic diseases. *Scandinavian Journal of Medicine & Science in Sports*.

[44] Eijsvogels TMH, Thompson PD (2015). Exercise Is Medicine: At Any Dose?. *JAMA*.

[45] Mons U, Hahmann H, Brenner H (2014). A reverse J-shaped association of leisure time physical activity with prognosis in patients with stable coronary heart disease: evidence from a large cohort with repeated measurements. *Heart (British Cardiac Society)*.

[46] Galiuto L, Liuzzo G (2022). Volume of physical activity and cardiovascular health status: is more necessarily better?. *European Heart Journal*.

[47] Bull FC, Al-Ansari SS, Biddle S, Borodulin K, Buman MP, Cardon G (2020). World Health Organization 2020 guidelines on physical activity and sedentary behaviour. *British Journal of Sports Medicine*.

[48] Zhang X, Lui J, Wu C, Cui J, Wu Y, Hu A (2021). Healthy lifestyle behaviours and all-cause and cardiovascular mortality among 0.9 million Chinese adults. *The International Journal of Behavioral Nutrition and Physical Activity*.

