# Supplementary material for: Time-Varying Effect of Physical Activity on Mortality Among Myocardial Infarction Survivors: A Nationwide Population-Based Cohort Study
Source: Rev Cardiovasc Med. 2023 Feb 22;24(3):67. doi: 10.31083/j.rcm2403067 (PMC11264011; doi:10.31083/j.rcm2403067)
Supplement: Supplementary file 1 [file 2153-8174-24-3-067-s1.docx]

**Supplementary File**

**Supplementary Section 1. Detailed information about sampling selection**

From August 2014 to December 2021, permanent residents aged 35-75 years that had a history of myocardial infarction were identified from 349 rural counties or urban districts (210 rural counties, 139 urban districts) in 31 provinces in mainland China in the China Patient-centered Evaluative Assessment of Cardiac Events (PEACE) Million Persons Project (MPP). The project sites (rural counties and urban districts) were selected based on their geographic locations within each province, the number of residents living in the rural or urban area, minority ethnicity distribution, quality of disease and death registries, and local capacity to support the project. Specifically, staff in the provincial coordinating office collected basic information (geographic information, economic development, population size, and minority ethnicity distribution) about the selected sites in their province; reported this information to the national coordinating office; and discussed it with staff in the national coordinating office to determine the study sites. In each site, about 8-9 towns or sub-districts were chosen according to their population size, population stability (e.g., no sudden significant change in the number of residents), local staff’s commitment, and ability to perform the screening. Initial screening stations were set up in each town or sub-district health center.

Potentially eligible participants were identified in each town or sub-district through official residential records and then invited by local community workers via telephone or through extensive publicity campaigns on television and in newspaper. All participants were required to bring their identity cards to the screening clinics to verify that they met the inclusion criteria: 1) aged 35 to 75 years; 2) registered in the selected site’s Hukou (a record officially identifying a person as a resident of an area), and lived in the selected regions at least 6 months during the last 12 months. After the verification, eligible participants who had signed the informed consent agreement were then enrolled in the project [1].

**Supplementary Section 2. Measurement of physical activity**

To quantify the volume of physical activity (PA), we asked the duration spent across all domains of life each day (leisure time, household, transport, and occupational domains). By multiplying the duration of PA by the metabolic equivalent of task (MET) value of each activity, and summing the MET minutes of all activities together, the volume of PA was obtained (MET minutes/week). The MET values of typical activities were calculated according to the updated 2011 Compendium of Physical Activities [2] and the fact of Chinese characteristics (below Table).

**Physical activity types, intensity categories, and MET values**

| **Activity type** | **Intensity** | **MET** |
| --- | --- | --- |
| Heavy manual work | Vigorous | 6.5 |
| Manual work | Moderate | 4.5 |
| Standing work | Moderate | 3·8 |
| Sedentary work | Low | 1·8 |
| Manual work in the farming season | Vigorous | 6·3 |
| Semi-mechanized work in the farming season | Moderate | 3·4 |
| Fully mechanized work in the farming season | Low | 2·4 |
| Work outside the farming season | Low | 2.0 |
| Walking | Moderate | 4.0 |
| Bicycle | Vigorous | 6·8 |
| Motorbike | Moderate | 3·5 |
| Private or public transportation (such as bus, car, underground, and ferry) | Low | 1·7 |
| Household activity | Low | 2·8 |
| Tai-Chi/qigong/leisure walking | Moderate | 3·3 |
| Jogging/aerobic exercise | Vigorous | 7·4 |
| Ball games | Moderate | 5·5 |
| Brisk walking/gymnastics/folk dancing | Moderate | 4·2 |
| Swimming | Vigorous | 7·2 |
| Other exercise, i.e. mountain walking, home exercise and rope jumping | Moderate | 5·9 |

MET: Metabolic equivalent of task

**Supplementary Section 3. Measurement of covariates**

Following 5 minutes of rest in a seated position, the mean of 2 blood pressure (BP) measurements was obtained using a standardized electronic BP monitor (Omron HEM-7430, Omron Corporation, Kyoto, Japan). If the difference between the 2 systolic BP readings was greater than 10mmHg, we took a third measurement and used the average of the last 2 readings. High BP was defined as systolic BP ≥140mmHg or diastolic BP ≥90mmHg. A non-fasting blood test that measures blood cholesterol (CardioChek PA Analyzer; Polymer Technology Systems, Indianapolis, Indiana, USA) and blood glucose (BeneCheck BK6-20M Multi-Monitoring System, Suzhou Pu Chun Tang Biotechnology Co. LTD, China) were performed by a standardized rapid analyzer using venous blood samples. High total cholesterol (TC) was defined as TC ≥5.0mmol/L. High glucose was defined as blood glucose ≥7.0mmol/L at least 8 hours after the last meal or random blood glucose ≥11.1mmol/L. Participants were required to wear light clothes, no shoes, and no cap when measuring height and weight. Body mass index (BMI) was defined as weight in kilograms divided by the square of height in meters. High BMI was defined as BMI ≥25kg/m^2^. We defined those who drank greater than 2 times per week as having high alcohol consumption. Participants were asked about smoking status (never, former, or current smokers). A food frequency questionnaire was used to collect dietary intake by asking about the frequency of consumption or typical food over the previous year. The participants were asked “During the past year, how often did you eat bellowing food”: daily, 4-6 days per week, 1-3 days per week, 1-3 days per month, never or almost never. This study included fresh fruit, fresh vegetable, bean and bean products, grains, and red meat. A healthy diet score was calculated (below Table). An unhealthy diet was defined as a healthy diet score of less than 4 points.

**The definition of healthy diet score**

| **Food components** | **“Healthy” (score=1)** |
| --- | --- |
| Fresh fruit | every day per week |
| Fresh vegetables | every day per week |
| Whole grains | every day per week |
| Fish and other seafood | ≥ 1 day per week |
| Bean and bean products | ≥ 4 days per week |
| Red meat | <7 days per week |

**References**

[1] Lu J, Xuan S, Downing NS, Wu C, Li L, Krumholz HM, et al. Protocol for the China PEACE (Patient-centered Evaluative Assessment of Cardiac Events) Million Persons Project pilot. BMJ Open. 2016; 6: e010200.

[2] Ainsworth BE, Haskell WL, Herrmann SD, Meckes N, Bassett DR, Jr., Tudor-Locke C, et al. 2011 Compendium of Physical Activities: a second update of codes and MET values. Med Sci Sports Exerc. 2011; 43: 1575-1581.

**
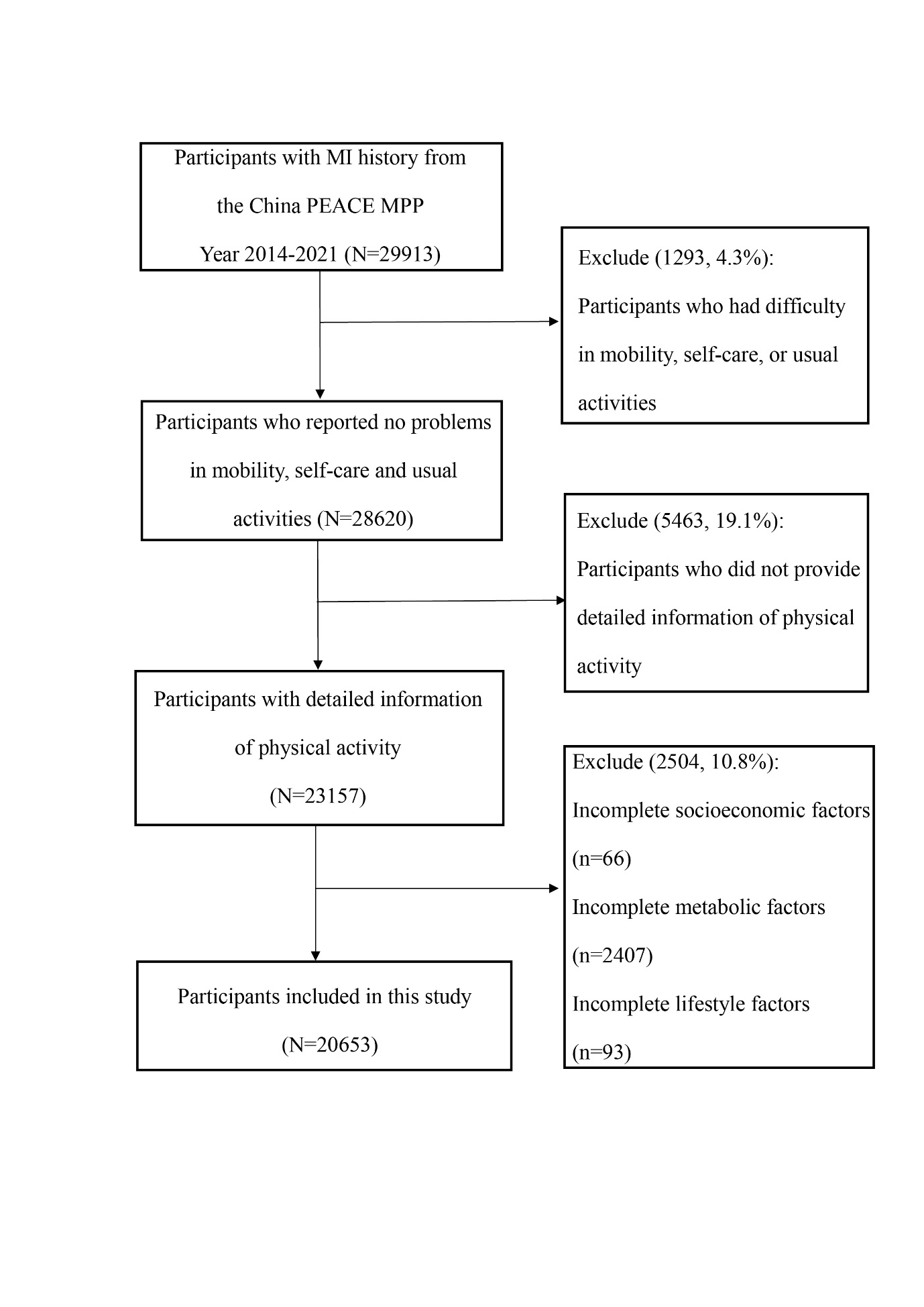
Supplementary Fig. 1. Study participants**

MI, myocardial infarction; China PEACE MPP, China Patient-centered Evaluative Assessment of Cardiac Events Million Persons Project.

**Supplementary Table 1. Baseline characteristics of the participants stratified by PA volumes and patterns**

| **Characteristics** | **Insufficient PA** | **Different volumes of sufficient PA** | | ***p-*value** | **Different patterns of sufficient PA** | | ***p-*value** |
| --- | --- | --- | --- | --- | --- | --- | --- |
|  |  | **Moderate** | **High** |  | **Leisure** | **Non-leisure** |  |
| Number of participants | 7805 (37.8%) | 3772 (18.3%) | 9076 (43.9%) |  | 2162 (10.5%) | 10686 (51.7%) |  |
| **Socioeconomic factors** |  |  |  |  |  |  |  |
| Age (years) | 64 [58, 69] | 64 [58, 68] | 60 [52, 66] | <0.001 | 64 [60, 68] | 60 [52, 66] | <0.001 |
| Sex |  |  |  |  |  |  |  |
| Female | 3428 (43.9%) | 1994 (52.9%) | 4432 (48.8%) | <0.001 | 947 (43.8%) | 5479 (51.3%) | <0.001 |
| Male | 4377 (56.1%) | 1778 (47.1%) | 4644 (51.2%) |  | 1215 (56.2%) | 5207 (48.7%) |  |
| Household income | | | | | | | |
| <10000 (yuan/year) | 1508 (19.3%) | 693 (18.4%) | 1679 (18.5%) | 0.533 | 222 (10.3%) | 2150 (20.1%) | <0.001 |
| ≥10000 (yuan/year) | 5773 (74.0%) | 2807 (74.4%) | 6760 (74.5%) |  | 1804 (83.4%) | 7763 (72.6%) |  |
| Unknown | 524 (6.7%) | 272 (7.2%) | 637 (7.0%) |  | 136 (6.3%) | 773 (7.2%) |  |
| Occupation type | |  |  |  |  |  |  |
| Light intensity | 353 (4.5%) | 245 (6.5%) | 2136 (23.5%) | <0.001 | 78 (3.6%) | 2303 (21.6%) | <0.001 |
| Medium or heavy intensity | 456 (5.8%) | 389 (10.3%) | 2128 (23.4%) |  | 38 (1.8%) | 2479 (23.2%) |  |
| Unemployed or retired | 6170 (79.1%) | 2650 (70.3%) | 2870 (31.6%) |  | 1958 (90.6%) | 3562 (33.3%) |  |
| Unknown | 826 (10.6%) | 488 (12.9%) | 1942 (21.4%) |  | 88 (4.1%) | 2342 (21.9%) |  |
| Education level |  |  |  |  |  |  |  |
| Primary school or below | 3273 (41.9%) | 1633 (43.3%) | 3651 (40.2%) | 0.003 | 670 (31.0%) | 4614 (43.2%) | <0.001 |
| Middle school or above | 4472 (57.3%) | 2114 (56.0%) | 5332 (58.7%) |  | 1478 (68.4%) | 5968 (55.8%) |  |
| Unknown | 60 (0.8%) | 25 (0.7%) | 93 (1.0%) |  | 14 (0.6%) | 104 (1.0%) |  |
| **Metabolic factors** |  |  |  |  |  |  |  |
| High blood pressure | 4052 (51.9%) | 1953 (51.8%) | 4414 (48.6%) | <0.001 | 1116 (51.6%) | 5251 (49.1%) | <0.001 |
| High blood glucose | 2075 (26.6%) | 959 (25.4%) | 2074 (22.9%) | <0.001 | 602 (27.8%) | 2431 (22.7%) | <0.001 |
| High total cholesterol | 2094 (26.8%) | 1083 (28.7%) | 2388 (26.3%) | 0.019 | 547 (25.3%) | 2924 (27.4%) | 0.137 |
| High body mass index | 4439 (56.9%) | 2065 (54.7%) | 4896 (53.9%) | 0.001 | 1243 (57.5%) | 5718 (53.5%) | <0.001 |
| **Lifestyle factors** |  |  |  |  |  |  |  |
| Current smoking | 1909 (24.5%) | 711 (18.8%) | 2169 (23.9%) | <0.001 | 418 (19.3%) | 2462 (23.0%) | <0.001 |
| High alcohol consumption | 809 (10.4%) | 319 (8.5%) | 999 (11.0%) | <0.001 | 224 (10.4%) | 1094 (10.2%) | 0.956 |
| Unhealthy diet | 6809 (87.2%) | 3279 (86.9%) | 7903 (87.1%) | 0.890 | 1733 (80.2%) | 9449 (88.4%) | <0.001 |
| **Medical History** |  |  |  |  |  |  |  |
| Heart failure | 394 (5.1%) | 189 (5.0%) | 365 (4.0%) | 0.003 | 103 (4.8%) | 451 (4.2%) | 0.027 |
| Chronic kidney disease | 77 (1.0%) | 36 (1.0%) | 75 (0.8%) | 0.524 | 30 (1.4%) | 81 (0.8%) | 0.013 |
| **Medication use** |  |  |  |  |  |  |  |
| ACEIs or ARBs | 972 (12.5%) | 476 (12.6%) | 993 (10.9%) | 0.002 | 323 (14.9%) | 1146 (10.7%) | <0.001 |
| Beta-blockers | 865 (11.1%) | 380 (10.1%) | 828 (9.1%) | <0.001 | 274 (12.7%) | 934 (8.7%) | <0.001 |
| Statins | 1273 (16.3%) | 563 (14.9%) | 1269 (14.0%) | <0.001 | 427 (19.8%) | 1405 (13.2%) | <0.001 |
| Aspirin | 1362 (17.5%) | 637 (16.9%) | 1443 (15.9%) | 0.024 | 439 (20.3%) | 1641 (15.4%) | <0.001 |

Data are presented as mean (SD), median [Q1, Q3 quartiles], or n (%) as appropriate.

ACEIs, angiotensin-converting enzyme inhibitors; ARBs, angiotensin receptor blockers; MET, metabolic equivalent of task; MI, myocardial infarction; PA, physical activity; SD, standard deviation.

**Supplementary Table 2. Summary of death events by post-MI periods and PA**

| **Total PA**  **(MET minutes/week)** | **Total period** | **Within 1 year of MI onset** | **Beyond 1 year of MI onset** |
| --- | --- | --- | --- |
| **All-cause** | 751 | 213 | 538 |
| Volumes |  |  |  |
| Insufficient (<3000) | 374 | 104 | 270 |
| Moderate (3000-4500) | 130 | 32 | 98 |
| High (>4500) | 247 | 77 | 170 |
| Patterns |  |  |  |
| Insufficient (<3000) | 374 | 104 | 270 |
| Non-leisure (main domain) | 313 | 96 | 217 |
| Leisure (main domain) | 64 | 13 | 51 |
|  |  |  |  |
| **Cardiovascular** | 446 | 127 | 319 |
| Volumes |  |  |  |
| Insufficient (<3000) | 229 | 70 | 159 |
| Moderate (3000-4500) | 80 | 21 | 59 |
| High (>4500) | 137 | 36 | 101 |
| Patterns |  |  |  |
| Insufficient (<3000) | 229 | 70 | 159 |
| Non-leisure (main domain) | 179 | 50 | 129 |
| Leisure (main domain) | 38 | 7 | 31 |

Total PA (MET minutes/week) was categorized as: insufficient (<3000); moderate (3000-4500) and high (>4500) volumes; non-leisure (sufficient PA with more input from non-leisure time PA) and leisure (sufficient PA with more input from leisure time PA) patterns.

**Supplementary Table 3. HRs (95% CI) for all-cause mortality and cardiovascular mortality by PA volumes at different post-MI periods**

| **Total PA**  **(MET minutes/week)** | **Total period** | | **Within 1 year of MI onset** | | **Beyond 1 year of MI onset** | | ***p*-interaction** |
| --- | --- | --- | --- | --- | --- | --- | --- |
|  | **HR (95% CI)** | ***p-*value** | **HR (95% CI)** | ***p-*value** | **HR (95% CI)** | ***p-*value** |  |
| **Unadjusted model** |  |  |  |  |  |  | 0.405 |
| Insufficient (<3000) | 1.00 |  | 1.00 |  | 1.00 |  |  |
| Moderate (3000-4500) | 0.73 (0.60-0.89) | 0.002 | 0.59 (0.40-0.88) | 0.009 | 0.79 (0.62-0.99) | 0.042 |  |
| High (>4500) | 0.71 (0.61-0.84) | <0.001 | 0.74 (0.55-1.00) | 0.050 | 0.70 (0.58-0.85) | <0.001 |  |
| **Multivariable-adjusted model** |  |  |  |  |  |  | 0.515 |
| Insufficient (<3000) | 1.00 |  | 1.00 |  | 1.00 |  |  |
| Moderate (3000-4500) | 0.77 (0.63-0.94) | 0.010 | 0.59 (0.40-0.88) | 0.011 | 0.83 (0.66-1.05) | 0.118 |  |
| High (>4500) | 0.68 (0.57-0.81) | <0.001 | 0.63 (0.45-0.88) | 0.008 | 0.69 (0.56-0.86) | 0.001 |  |

1. **All-cause mortality**
2. **Cardiovascular mortality**

| **Total PA**  **(MET minutes/week)** | **Total period** | | **Within 1 year of MI onset** | | **Beyond 1 year of MI onset** | | ***p*-interaction** |
| --- | --- | --- | --- | --- | --- | --- | --- |
|  | **HR (95% CI)** | ***p-*value** | **HR (95% CI)** | ***p-*value** | **HR (95% CI)** | ***p-*value** |  |
| **Unadjusted model** |  |  |  |  |  |  | 0.236 |
| Insufficient (<3000) | 1.00 |  | 1.00 |  | 1.00 |  |  |
| Moderate (3000-4500) | 0.73 (0.57-0.94) | 0.016 | 0.58 (0.35-0.94) | 0.026 | 0.80 (0.59-1.08) | 0.141 |  |
| High (>4500) | 0.63 (0.51-0.78) | <0.001 | 0.51 (0.34-0.78) | 0.002 | 0.68 (0.53-0.87) | 0.003 |  |
| **Multivariable-adjusted model** |  |  |  |  |  |  | 0.291 |
| Insufficient (<3000) | 1.00 |  | 1.00 |  | 1.00 |  |  |
| Moderate (3000-4500) | 0.78 (0.60-1.01) | 0.058 | 0.59 (0.36-0.97) | 0.038 | 0.85 (0.63-1.14) | 0.273 |  |
| High (>4500) | 0.59 (0.46-0.75) | <0.001 | 0.45 (0.29-0.72) | 0.001 | 0.64 (0.48-0.84) | 0.002 |  |

Total PA (MET minutes/week) was categorized as: insufficient (<3000), moderate (3000-4500) and high (>4500) volumes.

Multivariable-adjusted model: HRs were adjusted for age, sex, household income, occupation type, education level, high blood pressure, high blood glucose, high total cholesterol, high body mass index, high alcohol consumption, current smoking, unhealthy diet, history of heart failure, history of chronic kidney disease, and medication use including angiotensin-converting enzyme inhibitors or angiotensin receptor blockers, beta-blockers, statins, and aspirin.

CI, confidence interval; HR, hazard ratio; MET, metabolic equivalent of task; MI, myocardial infarction; PA, physical activity.

**Supplementary Table 4. HRs (95% CI) for all-cause mortality and cardiovascular mortality by PA volumes at different post-MI periods (Sensitivity analyses: using inverse probability weighting method)**

| **Total PA**  **(MET minutes/week)** | **Total period** | | **Within 1 year of MI onset** | | **Beyond 1 year of MI onset** | | ***p*-interaction** |
| --- | --- | --- | --- | --- | --- | --- | --- |
|  | **HR (95% CI)** | ***p-*value** | **HR (95% CI)** | ***p-*value** | **HR (95% CI)** | ***p-*value** |  |
| **Unadjusted model** |  |  |  |  |  |  | 0.422 |
| Insufficient (<3000) | 1.00 |  | 1.00 |  | 1.00 |  |  |
| Moderate (3000-4500) | 0.75 (0.62-0.92) | 0.004 | 0.62 (0.42-0.91) | 0.015 | 0.80 (0.64-1.00) | 0.054 |  |
| High (>4500) | 0.71 (0.61-0.82) | <0.001 | 0.72 (0.55-0.95) | 0.018 | 0.70 (0.58-0.83) | <0.001 |  |
| **Multivariable-adjusted model** |  |  |  |  |  |  | 0.536 |
| Insufficient (<3000) | 1.00 |  | 1.00 |  | 1.00 |  |  |
| Moderate (3000-4500) | 0.75 (0.62-0.91) | 0.004 | 0.59 (0.40-0.88) | 0.009 | 0.81 (0.64-1.01) | 0.059 |  |
| High (>4500) | 0.68 (0.58-0.80) | <0.001 | 0.63 (0.47-0.86) | 0.003 | 0.69 (0.57-0.83) | <0.001 |  |

1. **All-cause mortality**
2. **Cardiovascular mortality**

| **Total PA**  **(MET minutes/week)** | **Total period** | | **Within 1 year of MI onset** | | **Beyond 1 year of MI onset** | | ***p*-interaction** |
| --- | --- | --- | --- | --- | --- | --- | --- |
|  | **HR (95% CI)** | ***p-*value** | **HR (95% CI)** | ***p-*value** | **HR (95% CI)** | ***p-*value** |  |
| **Unadjusted model** |  |  |  |  |  |  | 0.300 |
| Insufficient (<3000) | 1.00 |  | 1.00 |  | 1.00 |  |  |
| Moderate (3000-4500) | 0.78 (0.61-1.00) | 0.048 | 0.61 (0.37-1.00) | 0.048 | 0.84 (0.63-1.12) | 0.243 |  |
| High (>4500) | 0.68 (0.56-0.83) | <0.001 | 0.57 (0.40-0.82) | 0.002 | 0.72 (0.58-0.91) | 0.005 |  |
| **Multivariable-adjusted model** |  |  |  |  |  |  | 0.358 |
| Insufficient (<3000) | 1.00 |  | 1.00 |  | 1.00 |  |  |
| Moderate (3000-4500) | 0.78 (0.61-1.00) | 0.051 | 0.59 (0.36-0.97) | 0.038 | 0.84 (0.63-1.13) | 0.249 |  |
| High (>4500) | 0.65 (0.53-0.80) | <0.001 | 0.51 (0.34-0.77) | 0.001 | 0.69 (0.54-0.89) | 0.003 |  |

Total PA (MET minutes/week) was categorized as: insufficient (<3000), moderate (3000-4500) and high (>4500) volumes.

Multivariable-adjusted model: HRs were adjusted for age, sex, household income, occupation type, education level, high blood pressure, high blood glucose, high total cholesterol, high body mass index, high alcohol consumption, current smoking, unhealthy diet, history of heart failure, history of chronic kidney disease, and medication use including angiotensin-converting enzyme inhibitors or angiotensin receptor blockers, beta-blockers, statins, and aspirin.

CI, confidence interval; HR, hazard ratio; MET, metabolic equivalent of task; MI, myocardial infarction; PA, physical activity.

**Supplementary Table 5. HRs (95% CI) for all-cause mortality and cardiovascular mortality by PA patterns at different post-MI periods**

| **Total PA**  **(MET minutes/week)** | **Total period** | | **Within 1 year of MI onset** | | **Beyond 1 year of MI onset** | | ***p*-interaction** |
| --- | --- | --- | --- | --- | --- | --- | --- |
|  | **HR (95% CI)** | ***p-*value** | **HR (95% CI)** | ***p-*value** | **HR (95% CI)** | ***p-*value** |  |
| **Unadjusted model** |  |  |  |  |  |  | 0.665 |
| Insufficient (<3000) | 1.00 |  | 1.00 |  | 1.00 |  |  |
| Non-leisure (main domain) | 0.76 (0.66-0.89) | <0.001 | 0.74 (0.56-0.98) | 0.035 | 0.77 (0.64-0.92) | 0.004 |  |
| Leisure (main domain) | 0.57 (0.44-0.74) | <0.001 | 0.46 (0.26-0.82) | 0.008 | 0.61 (0.46-0.83) | 0.001 |  |
| **Multivariable-adjusted model** |  |  |  |  |  |  | 0.775 |
| Insufficient (<3000) | 1.00 |  | 1.00 |  | 1.00 |  |  |
| Non-leisure (main domain) | 0.75 (0.64-0.89) | 0.001 | 0.64 (0.46-0.88) | 0.006 | 0.79 (0.65-0.97) | 0.024 |  |
| Leisure (main domain) | 0.61 (0.47-0.80) | <0.001 | 0.52 (0.29-0.94) | 0.029 | 0.64 (0.48-0.87) | 0.004 |  |

1. **All-cause mortality**
2. **Cardiovascular mortality**

| **Total PA**  **(MET minutes/week)** | **Total period** | | **Within 1 year of MI onset** | | **Beyond 1 year of MI onset** | | ***p*-interaction** |
| --- | --- | --- | --- | --- | --- | --- | --- |
|  | **HR (95% CI)** | ***p-*value** | **HR (95% CI)** | ***p-*value** | **HR (95% CI)** | ***p-*value** |  |
| **Unadjusted model** |  |  |  |  |  |  | 0.198 |
| Insufficient (<3000) | 1.00 |  | 1.00 |  | 1.00 |  |  |
| Non-leisure (main domain) | 0.70 (0.57-0.85) | <0.001 | 0.58 (0.40-0.84) | 0.004 | 0.75 (0.59-0.94) | 0.014 |  |
| Leisure (main domain) | 0.55 (0.39-0.78) | <0.001 | 0.37 (0.17-0.80) | 0.011 | 0.63 (0.43-0.93) | 0.020 |  |
| **Multivariable-adjusted model** |  |  |  |  |  |  | 0.268 |
| Insufficient (<3000) | 1.00 |  | 1.00 |  | 1.00 |  |  |
| Non-leisure (main domain) | 0.68 (0.55-0.85) | 0.001 | 0.53 (0.35-0.80) | 0.003 | 0.74 (0.57-0.96) | 0.025 |  |
| Leisure (main domain) | 0.61 (0.43-0.86) | 0.005 | 0.43 (0.20-0.94) | 0.033 | 0.67 (0.45-0.99) | 0.043 |  |

Total PA (MET minutes/week) was categorized as: insufficient (<3000), non-leisure (sufficient PA with more input from non-leisure time PA) and leisure (sufficient PA with more input from leisure time PA) patterns.

Multivariable-adjusted model: HRs were adjusted for age, sex, household income, occupation type, education level, high blood pressure, high blood glucose, high total cholesterol, high body mass index, high alcohol consumption, current smoking, unhealthy diet, history of heart failure, history of chronic kidney disease, and medication use including angiotensin-converting enzyme inhibitors or angiotensin receptor blockers, beta-blockers, statins, and aspirin.

CI, confidence interval; HR, hazard ratio; MET, metabolic equivalent of task; MI, myocardial infarction; PA, physical activity.

**Supplementary Table 6. HRs (95% CI) for all-cause mortality and cardiovascular mortality by PA patterns at different post-MI periods (Sensitivity analyses: using inverse probability weighting method)**

| **Total PA**  **(MET minutes/week)** | **Total period** | | **Within 1 year of MI onset** | | **Beyond 1 year of MI onset** | | ***p*-interaction** |
| --- | --- | --- | --- | --- | --- | --- | --- |
|  | **HR (95% CI)** | ***p-*value** | **HR (95% CI)** | ***p-*value** | **HR (95% CI)** | ***p-*value** |  |
| **Unadjusted model** |  |  |  |  |  |  | 0.651 |
| Insufficient (<3000) | 1.00 |  | 1.00 |  | 1.00 |  |  |
| Non-leisure (main domain) | 0.75 (0.65-0.86) | <0.001 | 0.70 (0.54-0.90) | 0.007 | 0.76 (0.65-0.90) | 0.001 |  |
| Leisure (main domain) | 0.52 (0.39-0.69) | <0.001 | 0.46 (0.25-0.83) | 0.010 | 0.55 (0.40-0.75) | <0.001 |  |
| **Multivariable-adjusted model** |  |  |  |  |  |  | 0.674 |
| Insufficient (<3000) | 1.00 |  | 1.00 |  | 1.00 |  |  |
| Non-leisure (main domain) | 0.75 (0.64-0.87) | <0.001 | 0.60 (0.45-0.81) | <0.001 | 0.79 (0.66-0.95) | 0.010 |  |
| Leisure (main domain) | 0.52 (0.40-0.69) | <0.001 | 0.46 (0.26-0.84) | 0.011 | 0.56 (0.41-0.77) | <0.001 |  |

1. **All-cause mortality**
2. **Cardiovascular mortality**

| **Total PA**  **(MET minutes/week)** | **Total period** | | **Within 1 year of MI onset** | | **Beyond 1 year of MI onset** | | ***p*-interaction** |
| --- | --- | --- | --- | --- | --- | --- | --- |
|  | **HR (95% CI)** | ***p-*value** | **HR (95% CI)** | ***p-*value** | **HR (95% CI)** | ***p-*value** |  |
| **Unadjusted model** |  |  |  |  |  |  | 0.231 |
| Insufficient (<3000) | 1.00 |  | 1.00 |  | 1.00 |  |  |
| Non-leisure (main domain) | 0.72 (0.60-0.86) | <0.001 | 0.58 (0.41-0.82) | 0.002 | 0.77 (0.62-0.96) | 0.017 |  |
| Leisure (main type) | 0.51 (0.36-0.74) | <0.001 | 0.41 (0.19-0.90) | 0.027 | 0.57(0.38-0.85) | 0.006 |  |
| **Multivariable-adjusted model** |  |  |  |  |  |  | 0.271 |
| Insufficient (<3000) | 1.00 |  | 1.00 |  | 1.00 |  |  |
| Non-leisure (main domain) | 0.71 (0.58-0.87) | 0.001 | 0.52 (0.35-0.77) | 0.001 | 0.77 (0.61-0.98) | 0.033 |  |
| Leisure (main domain) | 0.52 (0.36-0.75) | <0.001 | 0.41 (0.19-0.89) | 0.025 | 0.58 (0.39-0.87) | 0.009 |  |

Total PA (MET minutes/week) was categorized as: insufficient (<3000), non-leisure (sufficient PA with more input from non-leisure time PA) and leisure (sufficient PA with more input from leisure time PA) patterns.

Multivariable-adjusted model: HRs were adjusted for age, sex, household income, occupation type, education level, high blood pressure, high blood glucose, high total cholesterol, high body mass index, high alcohol consumption, current smoking, unhealthy diet, history of heart failure, history of chronic kidney disease, and medication use including angiotensin-converting enzyme inhibitors or angiotensin receptor blockers, beta-blockers, statins, and aspirin.

CI, confidence interval; HR, hazard ratio; MET, metabolic equivalent of task; MI, myocardial infarction; PA, physical activity.
